# Survival outcomes of abdominal radical hysterectomy, laparoscopic radical hysterectomy, robot-assisted radical hysterectomy and vaginal radical hysterectomy approaches for early-stage cervical cancer: a retrospective study

**DOI:** 10.1186/s12957-023-03051-4

**Published:** 2023-07-04

**Authors:** Nina Zhang, Xiangshu Jin, Wen Yang, Chenglei Gu, Li’an Li, Jia Xu, Qiting Tang, Wensheng Fan, Yuanguang Meng

**Affiliations:** Department of Obstetrics and Gynaecology, the Seventh Medical Centre of Chinese PLA General Hospital, Beijing, China

**Keywords:** Cervical cancer, ARH, LRH, RRH, VRH, Survival

## Abstract

**Background:**

This study compared the survival outcomes of abdominal radical hysterectomy (ARH) (*N* = 32), laparoscopic radical hysterectomy (LRH) (*N* = 61), robot-assisted radical hysterectomy (RRH) (*N* = 100) and vaginal radical hysterectomy (VRH) (*N* = 45) approaches for early-stage cervical cancer to identify the surgical approach that provides the best survival.

**Methods:**

Disease-free survival (DFS) and overall survival (OS) were calculated using the Kaplan–Meier method, and survival curves were compared using the log-rank test.

**Results:**

The volume of intraoperative blood loss was greater in the ARH group than in the LRH group, the RRH group or the VRH group [(712.50 ± 407.59) vs. (224.43 ± 191.89), (109.80 ± 92.98) and (216.67 ± 176.78) ml, respectively; *P* < 0.001]. Total 5-year OS was significantly different among the four groups (ARH, 96.88%; LRH, 82.45%; RRH, 94.18%; VRH, 91.49%; *P* = 0.015). However, no significant difference in 5-year DFS was observed among the four groups (ARH, 96.88%; LRH, 81.99%; RRH, 91.38%; VRH, 87.27%; *P* = 0.061).

**Conclusion:**

This retrospective study demonstrated that ARH and RRH achieved higher 5-year OS rates than LRH for early-stage cervical cancer.

## Background

Cervical cancer (CC) is one of the most important malignant tumours threatening women’s lives and health worldwide. In 2018, there were approximately 570,000 new cases of CC worldwide, and 311,000 patients died of this disease [1, 2]. Approximately 90% of CC-related deaths occur in developing countries, where the mortality rate is estimated to be 18 times as high as in developed countries [3]. The incidence of CC is 9.9/100,000 in developed countries, and the mortality is 3.3/100,000. Meanwhile, the incidence of CC is 15.7/100,000 in developing countries, and the mortality is 8.3/100,000 [4]. Early-stage CC is usually asymptomatic and can be detected by screening on physical examination. Most outpatient patients with cervical cancer have combined contact bleeding or abnormal vaginal bleeding and/or discharge [5]. Surgery and radiation therapy are primary treatments for CC, and both treatments are thought to achieve similar survival outcomes [6]. However, patients with early-stage CC are usually treated with radical hysterectomy [7].

Laparoscopic surgery became the standard approach for radical hysterectomy in 2014 [8]. Nevertheless, in the phase III Laparoscopic Approach to Cervical Cancer (LACC) trial, minimally invasive surgery (MIS) was associated with lower disease-free survival (DFS) and overall survival (OS) rates than open surgery in patients with early-stage CC [9, 10]. Conversely, current clinical data showed that there was no significant difference in survival outcomes between MIS and open surgery for patients with cervical cancer [11]. These recent findings are contradictory to earlier guidelines, which leads to wide controversy. Therefore, in this study, we summarized the case data of CC patients in a single centre over a span of 5 years (from January 2013 to December 2017) and evaluated the survival outcomes of four different surgical approaches, namely, abdominal, laparoscopic, robot-assisted and vaginal radical hysterectomy (ARH, LRH, RRH and VRH, respectively), for early-stage CC to define the benefits of the different radical hysterectomy approaches.

## Methods

### Patient enrolment

Patients with early-stage cervical cancer (Stage IA2-IB2) in the Department of Obstetrics and Gynaecology of the First Medical Center of Chinese PLA General Hospital (PLAGH) from January 2013 to December 2017 were analysed. All enrolled patients were treated with radical hysterectomy and grouped according to surgical approach. The patients fully understood the advantages and disadvantages of the various surgical treatments for CC before undergoing surgery and voluntarily chose the surgical approach.

### Inclusion criteria

The patients with cervical cancer were diagnosed by TCT, HPV, biopsy and/or conization. Patients had squamous cell carcinoma, adenocarcinoma or adenosquamous carcinoma of the uterine cervix. Patients were diagnosed as stage IA2 (stromal invasion, 3 to 5 mm in depth and < 7 mm in width), IB1 (tumour size of ≤ 4 cm in the greatest dimension) and IB2 (tumour size of > 4 cm in the greatest dimension) according to the 2009 FIGO (International Federation of Obstetrics and Gynaecology) staging system [12]. The patients underwent radical hysterectomy, including ARH, LRH, RRH and VRH, along with laparoscopic pelvic lymphadenectomy. Surgery, perioperative management, related clinical decision-making and postoperative follow-up were performed by the same medical team.

### Exclusion criteria

Patients treated with neoadjuvant therapy (chemotherapy or radiotherapy) were excluded. We also excluded patients with higher than stage IB2 disease, those who did not undergo radical hysterectomy, those who were generally in poor condition or had severe diseases and could not tolerate anaesthesia and surgery, those who had other malignant tumours or infectious diseases that were difficult to control, those who were lost to follow-up, those who died from causes not related to CC, and those with incomplete case data.

### Cohort selection

**Fig. 1:**
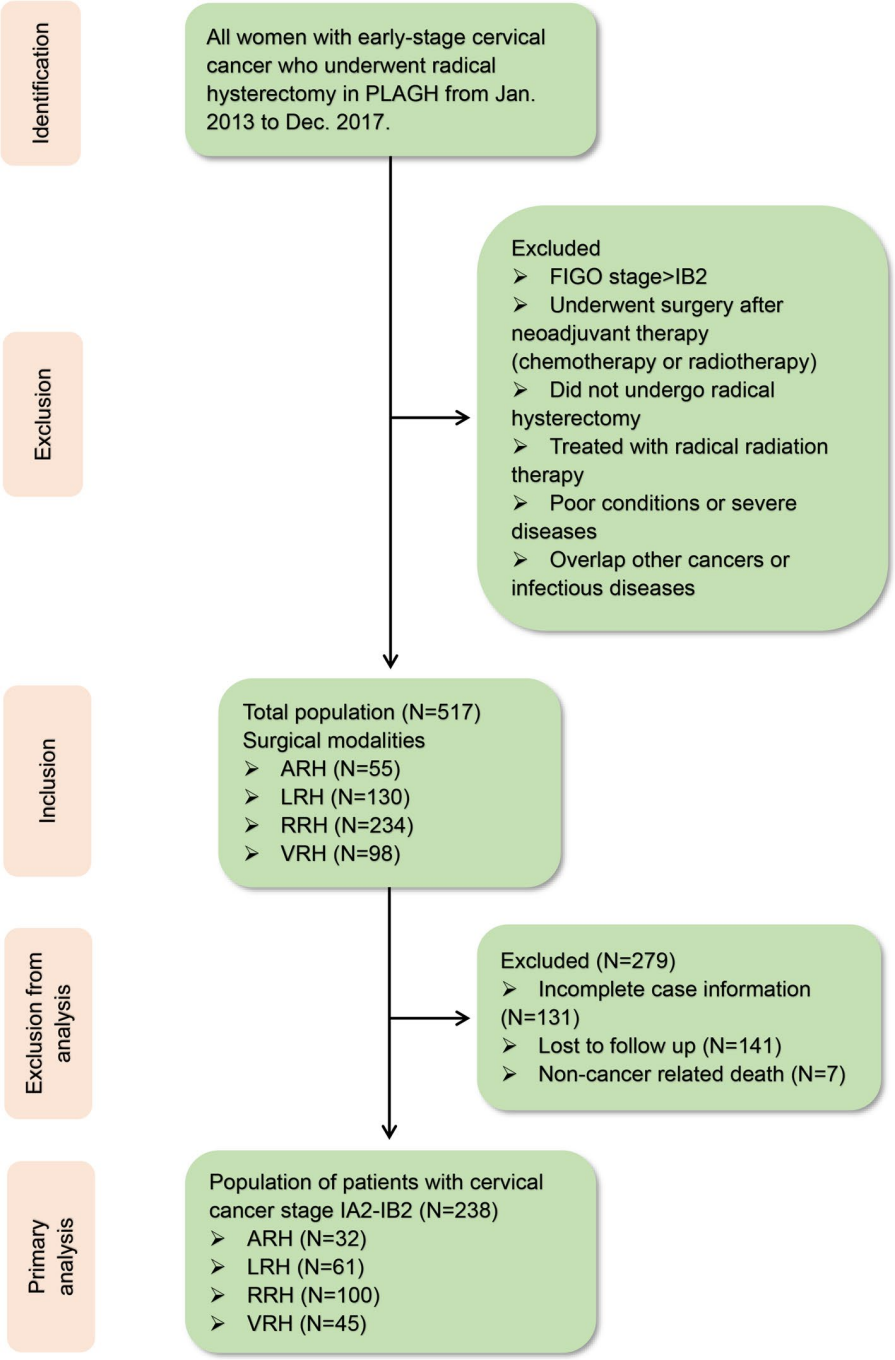
CONSORT (Consolidated Standards of Reporting Trials) diagram. Patients with early-stage cervical cancer who underwent radical surgery from January 2013 to December 2017

There were 517 patients diagnosed with stage IA2-IB2 CC in the First Medical Centre of PLAGH from January 2013 to December 2017. According to the above inclusion and exclusion criteria, 238 patients were enrolled (Fig. 1). Among them, 32 patients were included in the ARH group, 61 patients were included in the LRH group, 100 patients were included in the RRH group and 45 patients were included in the VRH group.

### Measures

The general information included age, body mass index (BMI), clinical stage and pathological type. The indicators examined in the perioperative period included intraoperative bleeding volume, operation time, blood transfusion rate, postoperative exhaust time, postoperative hospital stay, number of lymph nodes resected, number of positive lymph nodes, length of removed vaginal wall, healthcare costs incurred during hospitalization (surgery, chemotherapy, other drugs, etc.) and major complications. Ultimately, we analysed DFS and OS.

### Follow-up

An updated medical history and physical examination were recommended every 3 months for the first year, every 6 months for the following 2 years and then annually thereafter. The tests included routine blood tests, biochemistry, tumour biomarkers, vaginal stump TCT and HPV, chest X-ray/chest CT, pelvic and abdominal CT/MRI or gynaecological ultrasound, urinary ultrasound, ultrasound of the hepatobiliary pancreas and retroperitoneal lymph nodes, and PET-CT/MRI when the patients had suspected recurrence.

### Statistical analysis

SPSS 22.0 software was used for statistical analysis. The data are presented as the mean ± SD. One-way ANOVA was used for comparisons among the four groups. A two-sided *P*-value < 0.05 was considered statistically significant. The DFS and OS were graphed using GraphPad Prism 7.00 and calculated using the Kaplan–Meier method, and survival curves were compared using the log-rank test.

## Results

### Baseline comparison of the four groups according to different radical hysterectomy approaches

We identified 517 patients who underwent radical hysterectomy for early-stage CC during the inclusion period. Of these, 238 patients (40.03%) were selected for primary analysis (Fig. 1). The majority of the patients had stage IB1 disease (89.92%). The baseline characteristics are summarized in Table 1. Thirty-one patients (96.88%) in the ARH group had stage IB1 disease, of whom 26 patients (81.25%) had tumour histology indicating squamous cell carcinoma, and 16 patients (50.00%) received postoperative adjuvant therapy. Fifty-two patients (85.25%) with stage IB1 disease were included in the LRH group, of whom 49 patients (80.33%) had squamous cell carcinoma according to tumour histology, and 33 patients (54.10%) received postoperative adjuvant therapy. The RRH group included 91 (91.00%) stage IB1 patients, of whom 89 patients (89.00%) had squamous cell carcinoma according to tumour histology, and 55 patients (55.00%) received postoperative adjuvant therapy. The VRH group included 40 patients (88.89%) with stage IB1 disease, of whom 40 (88.89%) had squamous cell carcinoma according to tumour histology, and 22 patients (48.89%) received postoperative adjuvant therapy. However, there were no significant differences in age, BMI, FIGO stage, histology or postoperative adjuvant therapy among the four groups (*P* > 0.05).

**Table 1:** The baseline characteristics of patients with early-stage cervical cancer

Variables	ARH group	LRH group	RRH group	VRH group	*P* value
**No. of patients**	32	61	100	45	
**Age (years old)**	50.13 ± 8.89	48.97 ± 8.59	48.64 ± 9.89	46.04 ± 8.16	0.220
**BMI (kg/m**^**2**^**)**	25.42 ± 3.24	24.06 ± 2.81	24.12 ± 3.50	24.15 ± 3.23	0.210
**FIGO stage**					0.701
IA2	0 (0.00%)	5 (8.20%)	5 (5.00%)	3 (6.67%)	
IB1	31 (96.88%)	52 (85.24%)	91 (91.00%)	40 (88.89%)	
IB2	1 (3.12%)	4 (6.56%)	4 (4.00%)	2 (4.44%)	
**Histology**					0.444
Squamous cell carcinoma	26 (81.25%)	49 (80.33%)	89 (89.00%)	40 (88.89%)	
Adenocarcinoma	6 (18.75%)	11 (18.03%)	11 (11.00%)	4 (8.89%)	
Adenosquamous carcinoma	0 (0.00%)	1 (1.64%)	0 (0.00%)	1 (2.22%)	
**Postoperative adjuvant therapy**	16 (50.00%)	33 (54.10%)	55 (55.00%)	22 (48.89%)	0.894

*BMI* Body mass index in kg/cm^2^, *FIGO* International Federation for Gynaecology and Obstetrics

### Comparison of perioperative indices among the four groups

Then, we compared the perioperative indicators among the four groups. Our data showed that there was no significant difference in postoperative hospital stays, number of removed lymph nodes, number of positive lymph nodes and resected length of the vagina in the four groups (*P* > 0.05), while the differences in mean surgery time, intraoperative blood loss, postoperative exhaust time and healthcare costs were significantly different (*P* < 0.05); these data are summarized in Table 2.

**Table 2:** Characteristics of the perioperative periods in four groups

Variables	ARH group	LRH group	RRH group	VRH group	*P* value
No. of patients	32	61	100	45	
Mean surgery time (min)	182.31 ± 55.75	184.34 ± 35.31	212.32 ± 57.13	139.11 ± 36.54	< 0.001
Intraoperative blood loss (ml)	712.50 ± 407.59	224.43 ± 191.89	109.80 ± 92.98	216.67 ± 176.78	< 0.001
Postoperative hospital stays (day)	13.88 ± 4.41	12.26 ± 6.39	11.70 ± 4.84	11.51 + 3.29	0.150
Postoperative exhaust time (day)	2.28 ± 0.77	1.85 ± 0.70	2.11 ± 0.62	1.96 ± 0.60	0.013
Healthcare cost (× 10^4^, CNY)	4.54 ± 1.21	3.73 ± 1.41	6.66 ± 1.32	3.12 ± 1.09	< 0.001
No. of lymph nodes	22.91 ± 9.74	23.59 ± 9.22	23.69 ± 10.17	21.87 ± 8.64	0.739
No. of positive lymph nodes	0.03 ± 0.18	0.21 ± 1.54	0.37 ± 1.35	0.13 ± 0.46	0.467
Resected length of the vagina (cm)	1.83 ± 0.83	1.89 ± 0.70	1.61 ± 0.68	1.72 ± 0.74	0.094

Additionally, the mean surgery time in the ARH group was the shortest among the four different groups, whereas RRH had the longest. The ARH group had the largest intraoperative blood loss volume among the four different groups, while the RRH group had the least intraoperative blood loss. However, the postoperative exhaust time in the LRH group was the shortest while that in the ARH group was the longest. Among the four groups, the healthcare costs were the highest in the RRH group.

### Comparison of survival outcomes among four different surgical approaches

**Fig. 2:**
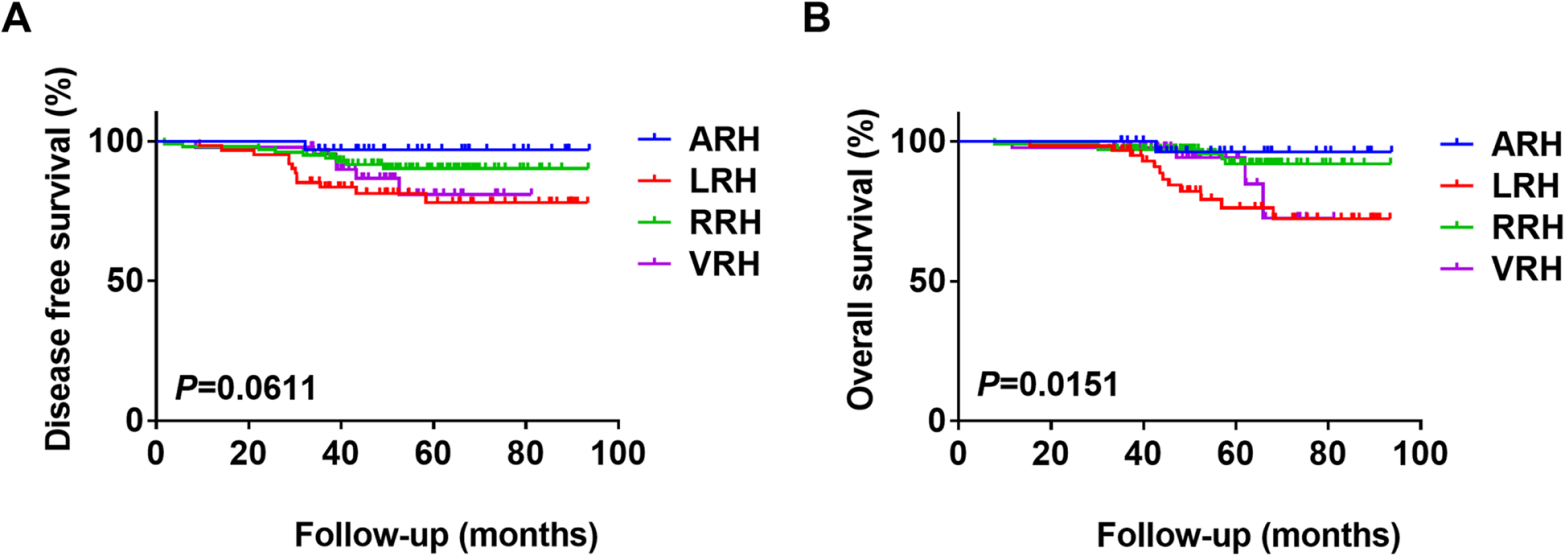
**A** Disease-free survival (DFS). **B** Overall survival (OS)

The mean follow-up time of all patients was 57 months (range 43 to 69 months), the interquartile range was 43 months to 68 months, the median follow-up time was 55 months (4.49 years), the 5-year DFS rate was 89.00% (95% CI 88.21–89.81%) and the 5-year OS rate was 91.13% (95% CI 90.07–92.20%). The five-year DFS rate was 96.88% in the ARH group, 81.99% (95% CI 83.21–86.56%) in the LRH group, 91.38% (95% CI 93.03–94.48%) in the RRH group and 87.27% (95% CI 87.85–91.47%) in the VRH group. The overall DFS curves of the four groups were compared using the log-rank test and showed no significant differences (*P* = 0.061) (Fig. 2A).

Furthermore, the 5-year OS rate was 96.88% (95% CI 96.38–97.37%) the ARH group, 82.45% (95% CI 87.17–92.00%) the LRH group, 94.18% (95% CI 96.95–97.23%) the RRH group and 91.49% (95% CI 95.42–96.74%) the VRH group. The overall OS curves of the four groups were compared using the log-rank test and showed statistically significant differences (*P* = 0.015) (Fig. 2B).

## Discussion

This study analysed the clinical data of patients with early-stage CC treated by four different surgical approaches (ARH, LRH, RRH and VRH) in a single centre over 5 years, and we noted no significant differences in DFS among the four groups. However, LRH was associated with shorter OS than ARH or RRH. Therefore, this study showed that not all the survival outcome indicators of MIS were inferior to those of ARH. Furthermore, the intraoperative blood loss and postoperative exhaust time of the three MIS procedures were better than those of ARH. The intraoperative blood loss was lowest in the RRH group, but this group had the highest healthcare costs.

This study is the first retrospective analysis to simultaneously compare the clinical characteristics and survival outcomes of ARH, LRH, RRH and VRH performed by the same medical team in a single centre. Based on our study results, we demonstrated that both ARH and RRH achieved higher 5-year OS than LRH for early-stage CC. Thus, we do not think that robotic surgery is unsafe as standard laparoscopic surgery. Nevertheless, the weakness of this study is the relatively small sample size of each group. We collected 517 patients to analyse the oncological outcomes of different radical hysterectomy approaches. However, based on the inclusive and exclusive criteria, we excluded approximately almost half of the whole data, which cannot allow us to draw definitive conclusions on the survival outcomes of different surgical approaches.

The standard approach for radical hysterectomy is the open abdominal approach. According to the guidelines, radical hysterectomy can be performed via open surgery and MIS. MIS has emerged as one of the preferred approaches for treating gynaecological malignancies [13, 14]. However, recent retrospective reviews and prospective observational studies demonstrated that MIS was associated with lower DFS and OS than open surgery in CC patients. The exact reason why MIS correlates with worse DFS and OS is still unknown. However, there are several potential explanations. These theories are as follows: (I) lower radically using MIS [15], (II) a lack of expertise in minimally invasive hysterectomy compared to open radical hysterectomy [16], (III) an increased risk of developing intraabdominal metastasis due to CO_2_ [17, 18], and (IV) tumour dissemination at the time of colpotomy [19]. Controversially, robot-assisted MIS obtained similar oncologic outcomes to open surgery. Therefore, the clinical advantages of robot-assisted MIS for the treatment of CC remain to be confirmed.

Since researchers reported the first case of laparoscopic radical cervical cancer [20], laparoscopic surgery and robotic surgery have been widely used in the treatment of CC patients and reported in many relevant clinical studies [21–23]. Most studies focused on perioperative conditions such as intraoperative blood loss, postoperative hospital stay, postoperative exhaust time and survival outcomes. A previous retrospective analysis showed that neither the laparoscopic approach nor the robot-assisted laparoscopic approach reduced the patients’ 5-year progression-free survival (PFS) and OS rates compared with the abdominal approach [24, 25]. Patients undergoing laparoscopy are at higher risk of developing intrapelvic recurrences and peritoneal carcinomatosis [26]. The LACC trial results showed that the 4.5-year PFS and 3-year OS rates in the MIS group were significantly lower than those in the ARH group, and the recurrence rate in early-stage CC patients who underwent MIS (84.4% laparoscopic and 15.6% robotic surgery) was approximately four times that in patients who underwent ARH [27]. Likewise, the SUCCOR study showed that MIS (78.5% laparoscopic and 21.5% robotic surgery) was associated with a lower OS rate than open surgery [28]. Nevertheless, the adoption of open surgery did not correlate with an increase in complication rates in those two analyses [27, 29]. In contrast, clinical data from the Memorial Sloan Kettering Cancer Centre showed that there was no significant difference in survival outcomes between MIS (10% laparoscopic and 90% robotic surgery) and open surgery for patients with CC, while the complication rates of MIS were significantly lower [11]. The adoption of robotic MIS does not seem to compromise survival when compared with open surgery [30]. The proportion of robotic surgery in MIS may be the key factor influencing the outcomes in these findings. Based on these results, compared with open surgery, MIS datasets comprising a low percentage of robotic surgery might lead to lower survival, whereas MIS datasets in which the majority of procedures are robotic surgery might lead to a lack of difference. Therefore, it is necessary to divide MIS into laparoscopic and robotic groups for comparisons with open surgery, as in our study. Moreover, conization may also affect the outcomes. Previous conization in patients undergoing radical hysterectomy was associated with improved DFS and OS compared with that in patients who did not undergo conization [31, 32]. At present, the factors that are most important for prognosis in CC are staging, tumour size, lymph node involvement, depth of stromal invasion and type of LVSI [33, 34]; additionally, the surgeon’s experience [35], the tumour free distance [36], the type of lymphadenectomy [37, 38] and the MIS approach should be adopted in selected cases [39, 40].

Currently, an international multicentre randomized controlled trial (Robot-Assisted Approach to Cervical Cancer, NCT03719547) evaluating the efficacy of robotic surgery and open surgery is underway in China [41]. In addition, although there is limited research on vaginal surgery for CC, it is still one of the surgical treatment options for patients with early-stage CC. A systematic review and meta-analysis showed that laparo-assisted vaginal radical hysterectomy did not appear to affect DFS and OS in early-stage CC patients, comparing with the open approach group of the LACC trial [42]. Nevertheless, MIS and vaginal surgery showed the highest recurrence rate compared to ARH for patients with early-stage CC regarding fertility-sparing treatments in tumours > 2 cm in size [43]. Thus, more studies will be needed to compare the surgical approaches of ARH, LRH, RRH and VRH in the early-stage CC.

In this study, we analysed the clinical data of patients with early-stage CC who underwent ARH, LRH, RRH and VRH performed by the same medical team in a single centre over 5 years and compared the perioperative indicators and survival outcomes. To date, this study is the newest analysis comparing the four different surgical approaches for patients with early-stage CC.

## Conclusions

In this retrospective study, we demonstrated that there was no significant difference in the mean age, BMI, FIGO stage, histology or postoperative adjuvant therapy among the ARH, LRH, RRH and VRH groups. The total 5-year OS curves were significantly different among the four groups. Additionally, the 5-year OS rates in the ARH and RRH groups were better than those in the LRH group. The survival outcomes of ARH and RRH were similar for patients with early-stage CC.

## Data Availability

Data are available from the corresponding author upon reasonable request.

## Abbreviations

ARH: Abdominal radical hysterectomy
BMI: Body mass index
CC: Cervical cancer
CONSORT: Consolidated Standards of Reporting Trials
DFS: Disease-free survival
FIGO: International Federation of Obstetrics and Gynaecology
LACC: Laparoscopic approach to cervical cancer
LRH: Laparoscopic radical hysterectomy
MIS: Minimally invasive surgery
OS: Overall survival
PFS: Progression-free survival
RRH: Robot-assisted radical hysterectomy
VRH: Vaginal radical hysterectomy
